# Supplementary material for: DNA-binding of the Tet-transactivator curtails antigen-induced lymphocyte activation in mice
Source: Nat Commun. 2017 Oct 18;8:1028. doi: 10.1038/s41467-017-01022-4 (PMC5647323; doi:10.1038/s41467-017-01022-4)
Supplement: Supplementary file 1 — Supplementary information [file 41467_2017_1022_MOESM1_ESM.pdf]

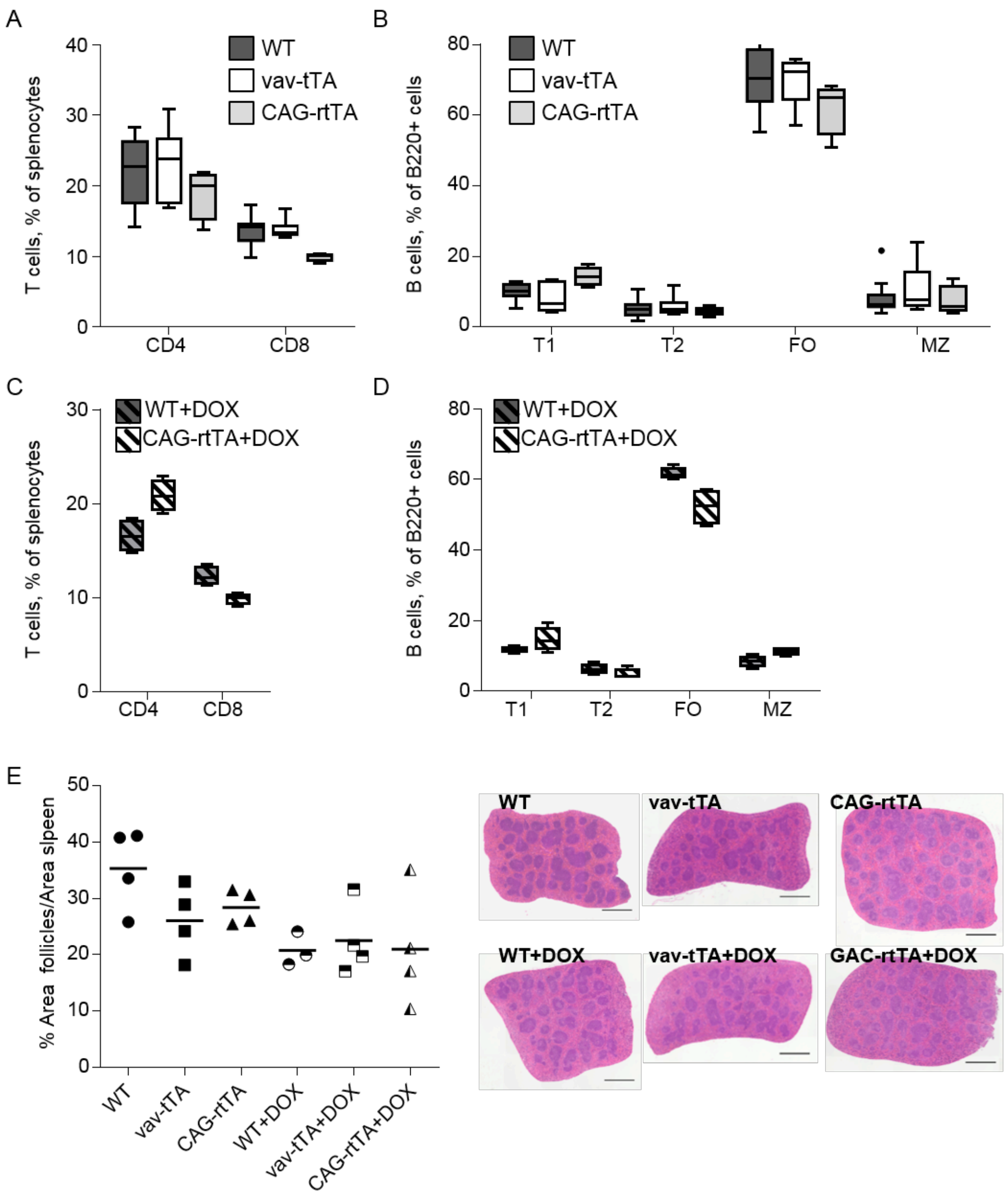

Supplementary Figure 1: vav-tTA and CAG-rtTA mice show normal lymphocyte subset composition in steady state.

Lymphocyte subsets and their precursors were quantified by flow-cytometric analysis of single cell suspensions from spleen of wt (n=11), vav-tTA (n=6) and CAG-rtTA mice (n=4), and wt (n=4) CAG-rtTA (n=4) mice fed with doxycycline-containing food for 7 days. **(A-C)** Percentages of $CD4^{+}$ and $CD8^{+}$ splenic cells **(B-D)** Percentages of transitional type 1 (T1) B cells ($B220^{high}$ IgM^{high} $CD23^{low}$ $CD21^{low}$), transitional type 2 (T2) B cells ($B220^{high}$ IgM^{high} $CD23^{+}$ $CD21^{high}$), follicular (FO) B cells ($B220^{high}$ IgM^{low} $CD23^{+}$ $CD21^{low}$) and marginal zone (MZ) B cells ($B220^{high}$ IgM^{high} $CD23^{low}$ $CD21^{high}$) gated as in Supplementary Figure 9a. Data are presented as box-and-whiskers-diagram with median and interquartile range, outliers are shown as dots. No significant differences were observed. **(E)** H&E stained spleen section from mice of the indicated genotypes kept ± doxycycline-containing food for 7 days. The predicted follicles area over total spleen area was calculated using the random forest classifier *ilastik*. Scale bars represent 1mm.

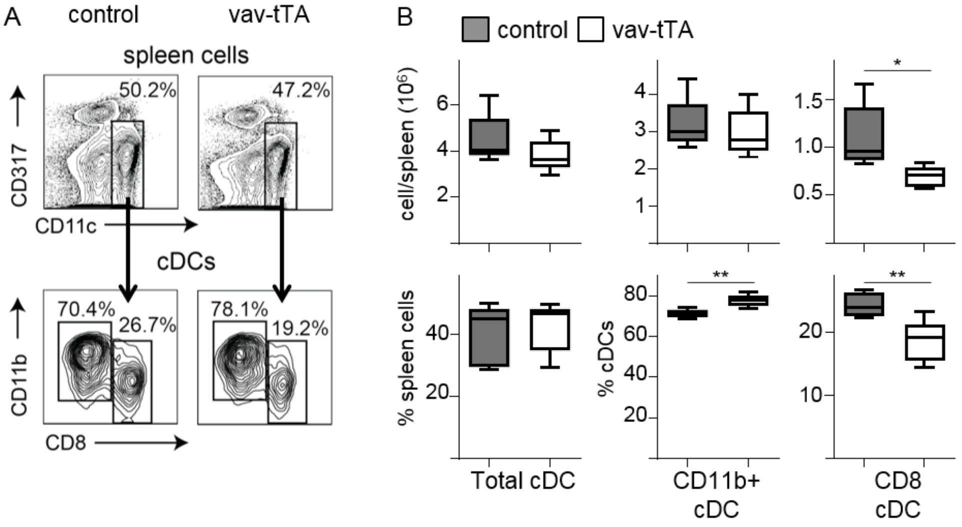

Supplementary Figure 2: Reduction of conventional CD8$^+$ dendritic cells in vav-tTA mice

Flow cytometric analysis of splenocytes from vav-tTA and controls (TRE-Ren) from two independent experiments and a total of 5 animals per genotype (n=5). **(A)** Representative staining with CD11c and CD317 to identify CD11c^{hi} CD317$^-$ conventional dendritic cells (cDCs), which were then further subdivided into CD11b$^+$ and CD8$^+$ subsets as shown in the figure. **(B)** Frequency and absolute cell numbers of total cDCs, CD11b$^+$ and CD8$^+$ cDCs Data are presented as box-and-whiskers-diagram with median and interquartile range. Absolute cell number was calculated by flow-cytometric analysis with the addition of a known number of Calbrite beads (BD). *$p < 0.05$, **$p < 0.01$, 2-tailed Mann-Whitney-U test.

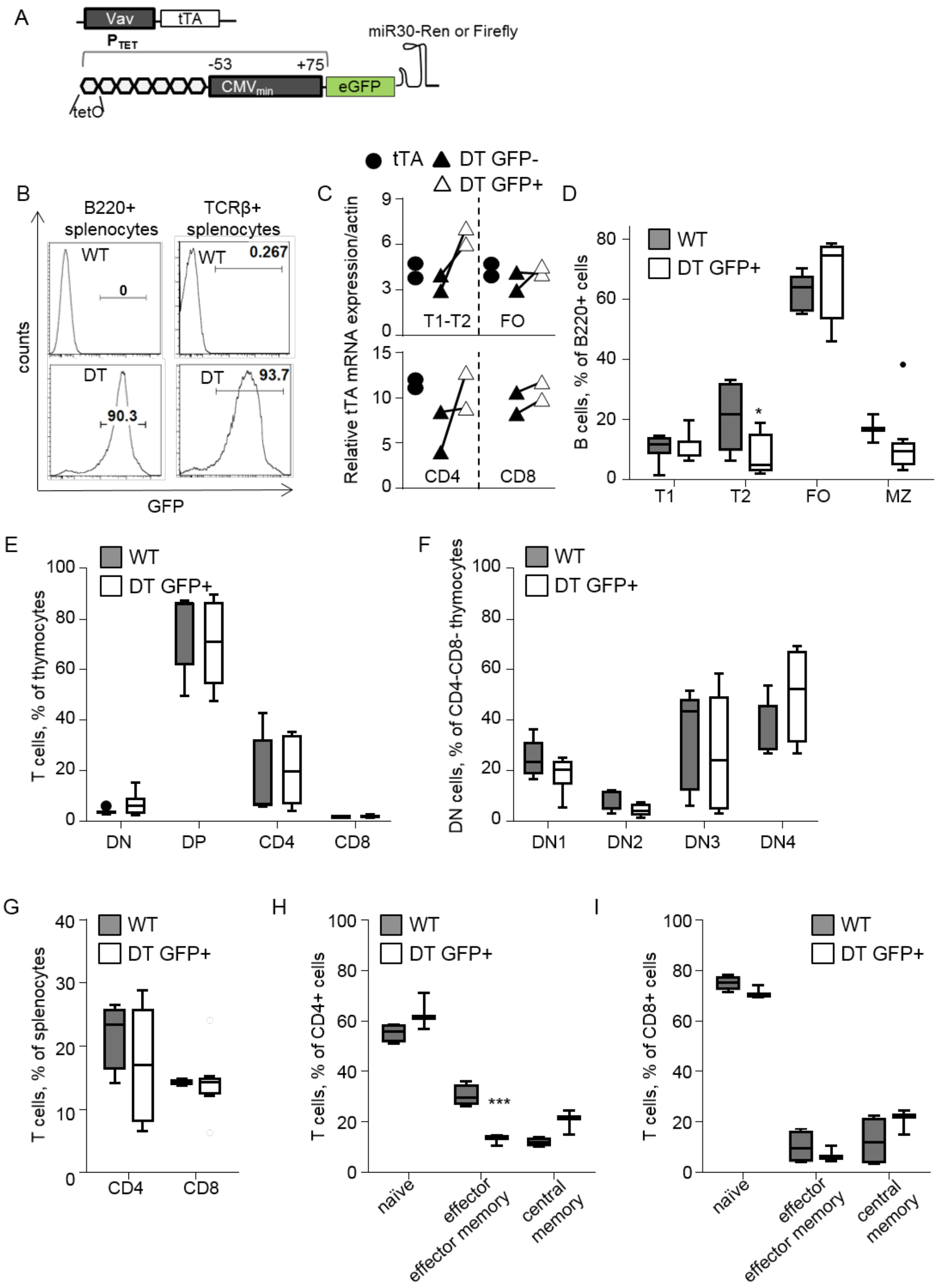

Supplementary Figure 3: tTA transgene expression does not impair lymphocyte composition in steady state

(A) Schematic representation of the transgenes expressed in the TRE_Ren/vav-tTA or TRE_Firefly/vav-tTA mice **(B)** GFP transgene expression in B220$^+$ B and TCRβ$^+$ T cells of TRE_Ren/vav-tTA (DT) and wt mice in steady state. **(C)** Relative tTA expression in sorted transitional (T1-T2) B cells (B220^{high} IgM^{high} IgD$^-$), follicular (FO) B cells (B220^{high} IgM^{low} IgD^{high}) mice, CD4$^+$ T cells and CD8$^+$ T cells from vav-tTA and DT mice. Each dot represents data from a single mouse as mean of results from assays in three technical replicates. The relative tTA mRNA expression was calculated as: $2^{(Actin\ CT\ -\ tTA\ CT)}$*10000. **(D)** Percentages of transitional type 1 (T1) B cells (B220^{high} IgM^{high} CD23^{low} CD21^{low}), transitional type 2 (T2) B

cells ($B220^{high}$ IgM^{high} $CD23^+$ $CD21^{high}$), follicular (FO) B cells ($B220^{high}$ IgM^{low} $CD23^+$ $CD21^{low}$) and marginal zone (MZ) B cells ($B220^{high}$ IgM^{high} $CD23^{low}$ $CD21^{high}$) in DT mice (n=5) and littermate controls (n=6) as gated in Supplementary Figure 9a. **(E)** Percentages of $CD4^-8^-$ double negative (DN), $CD4^+CD8^+$ double positive (DP) as well as $CD4^+CD8^-$ (CD4) and $CD4^-CD8^+$ (CD8) single positive thymocytes and **(F)** frequency of double negative (DN) thymocytes stages 1-4 ($CD25^-CD44^+$ DN1; $CD25^+CD44^+$ DN2; $CD25^+CD44^-$ DN3; $CD25^-CD44^-$ DN4), in DT mice (n=6) or littermate controls (n=7). **(G)** Frequencies of $CD4^+$ and $CD8^+$ splenic T cells and **(H)** naïve ($CD4^+$ $CD62L^{high}$ $CD44^-$), effector and effector memory ($CD4^+$ $CD62L^-$ $CD44^-$) and central memory ($CD4^+$ $CD62L^{high}$ $CD44^+$) $CD4^+$ T cells, or (**I**) naïve ($CD8^+$ $CD62L^{high}$ $CD44^-$), effector and effector memory ($CD8^+$ $CD62L^-$ $CD44^-$) and central memory ($CD8^+$ $CD62L^{high}$ $CD44^+$) $CD8^+$ T cells from DT mice (n=5) or littermate controls (n=4). Data are presented as box-and-whiskers-diagram with median and interquartile range, outliers are shown as dots. *$p < 0.05$, ***$p \leq 0.001$ GFP^+ vs. wild type controls, two-way ANOVA with Bonferroni correction for multiple comparisons.

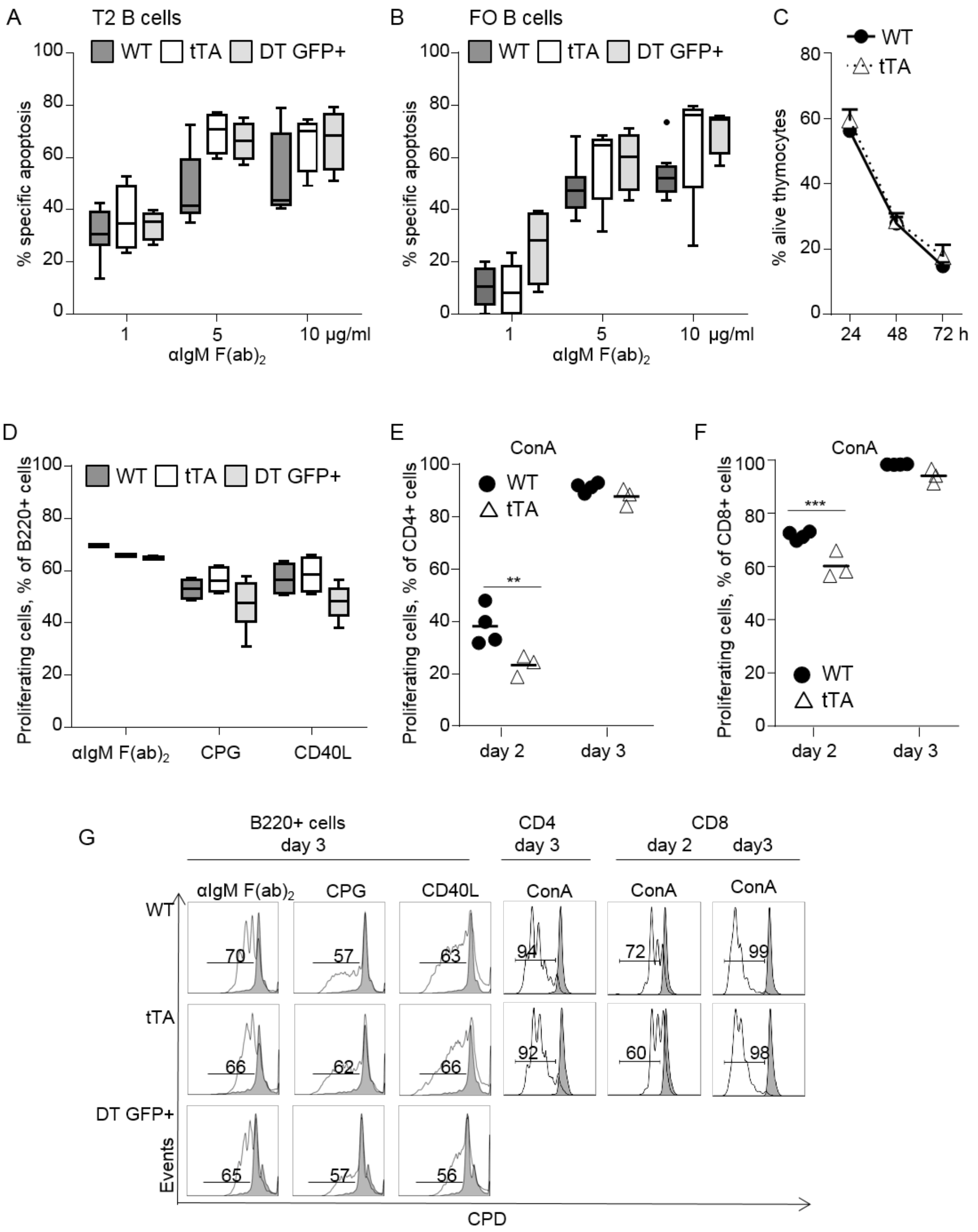

Supplementary Figure 4: Normal B cell proliferation and survival upon tTA expression in vitro

(**A**) Transitional type 2 (T2) ($B220^{high}$ IgM^{high} $CD23^{+}$ $CD21^{high}$), or **(B)** follicular (FO) B cells ($B220^{high}$ IgM^{low} $CD23^{+}$ $CD21^{low}$) from wild type (n=9), vav-tTA (n=4) and TRE_Ren/vav-tTA (DT) (n=4) mice were sorted based on GFP expression and cultured in the presence of graded concentrations of plate-bound αIgM F(ab)2 fragments. For the gating strategy see Supplementary Figure 9a. Cell viability was assessed at 18 hours post stimuli by 7AAD exclusion and flow cytometric analysis. The extent of apoptosis induced specifically by BCR ligation was calculated by the following equation: (induced apoptosis/spontaneous cell death)*100. Data are presented as box-and-whiskers-diagram with median and interquartile range, outliers are shown as dots. **(C)** Spontaneous survival of thymocytes in culture from wt (n=4) and vav-tTA (n=4) mice, living cells were quantified by TO-PRO3-Annexin-V exclusion. Data are presented as mean ± SD. No significant differences were observed. **(D)** Mitogen-induced proliferation of splenic $B220^{+}$ B lymphocytes from wild type (n=4), vav-tTA (n=4) and DT mice (n=5) treated with αIgM F(ab)2, αCD40 or

CpG. Mitogen induced proliferation of $CD4^+$ **(E)** and $CD8^+$ **(F)** splenic cells from vav-tTA (n=3) and wt (n=4) mice treated with the lectin Concavalin A (ConA) stimuli. Data are presented as box-and-whiskers-diagram with median and interquartile range or scatter plot. $**p \leq 0.01$, $***p \leq 0.001$ vav-tTA versus WT, two-way ANOVA with Bonferroni correction for multiple comparisons. **(G)** Representative histograms of treated lymphocytes derived from the mice of the indicated genotypes and GFP^+ reporter fractions (open histogram) compared to un-stimulated control cells (filled histograms).

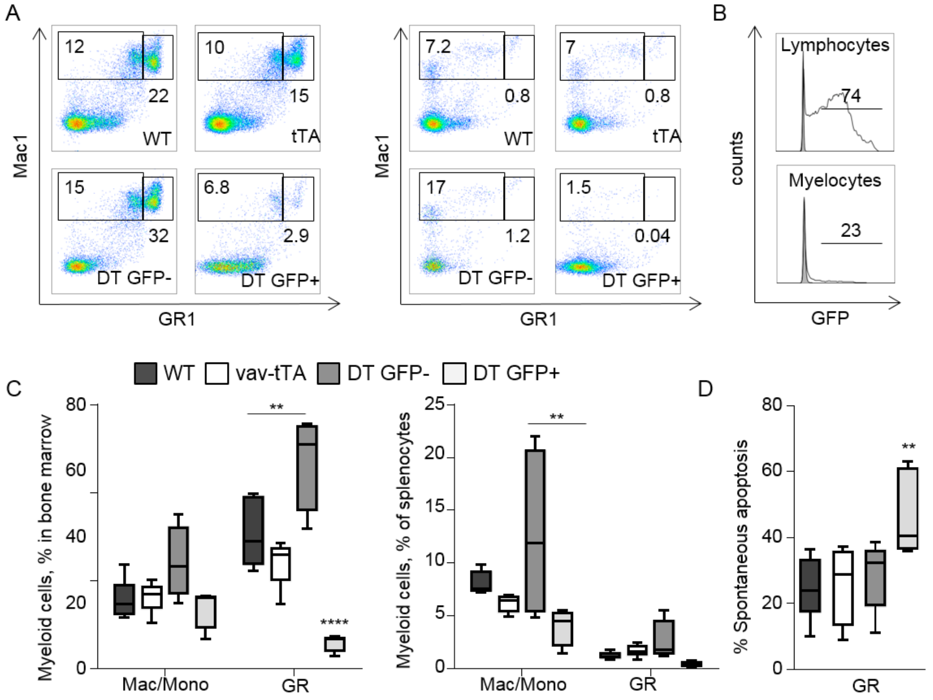

Supplementary Figure 5: tTA-driven GFP expression in myeloid cells correlates with impaired granulocyte survival.

(A) Representative dot blots and of flow cytometric analyses of bone marrow (left panel) and spleen (right panel) of wild type, vav-tTA and TRE_Ren/vav-tTA (DT) mice sorted on the basis of their GFP expression levels, using antibodies to identify the Mac-1$^+$Gr-1$^-$ monocytes/macrophages and the Mac-1$^+$Gr-1$^+$ granulocytes. **(B)** GFP transgene expression in lymphocytes (Mac1$^-$GR1$^-$) and myeloid cells (Mac1$^+$) cells in splenocyte from DT (open histogram) and wt mice (filled histogram). **(C)** Frequencies of Mac-1$^+$Gr-1$^-$ monocytes/macrophages and Mac-1$^+$Gr-1$^+$ granulocytes (GR) in bone marrow (left panel) and spleen (right panel) from of wild type (n=4), vav-tTA (n=4) and TRE_Ren/vav-tTA (DT) (n=4) mice. **p ≤ 0.01 GFP+ versus GFP- or wild type controls, two-way ANOVA with Bonferroni correction for multiple comparisons. **(D)** Survival analysis on sorted Mac-1$^+$Gr-1$^+$ granulocytes (GR) from wild type (n=8), vav-tTA (n=4) and DT (n=5) mice. Viability was assessed by 7AAD exclusion and flow cytometric analysis after 24 hours in culture. Data are presented as box-and-whiskers-diagram with median and interquartile range. **p ≤ 0.01 GFP+ versus GFP-, vav-tTA or wild type controls, one-way ANOVA.

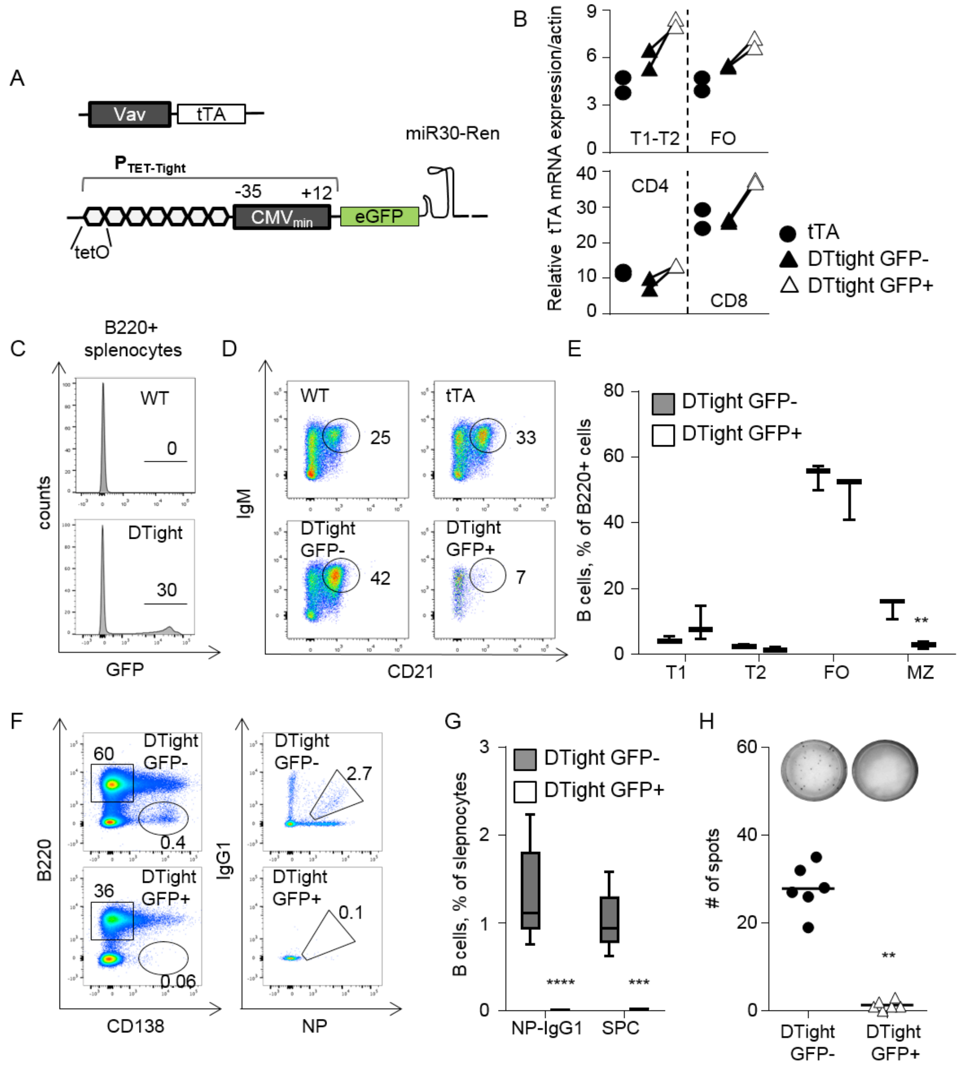

Supplementary Figure 6: tTA overexpression is responsible for impairment of B cell immunity.

(A) Schematic representation of the transgenes in the TRE_{tight}_Ren/vav-tTA mice (DTtight) mice. **(B)** Relative tTA mRNA expression assessed via qRT-PCR in sorted transitional transitional (T1-T2) B cells ($B220^{high}$ IgM^{high} IgD^{-}), follicular (FO) B cells ($B220^{high}$ IgM^{low} IgD^{high}), $CD4^{+}$ T cells and $CD8^{+}$ T cells from vav-tTA and DTtight mice. Each dot represents data from a single mouse as mean of results from assays performed in three technical replicates. The relative tTA mRNA expression was calculated as: $2^{(Actin\ CT\ -\ tTA\ CT)}*10000$. **(C)** GFP transgene expression in $B220^{+}$ B cells of DTtight and wt mice. **(D)** Representative dot plots of marginal zone (MZ) B cells ($B220^{high}$ IgM^{high} $CD23^{low}$ $CD21^{high}$) gated as in Supplementary Figure 9a and **(E)** quantification of transitional type 1 (T1) B cells ($B220^{high}$ IgM^{high} $CD23^{low}$ $CD21^{low}$), transitional type 2 (T2) B cells ($B220^{high}$ IgM^{high} $CD23^{+}$ $CD21^{high}$), follicular (FO) B cells ($B220^{high}$ IgM^{low} $CD23^{+}$ $CD21^{low}$) and MZ B cells in the GFP^{+} and GFP^{-} fraction of cells of the DTtight (n=6) mice. Data are presented as box-and-whiskers-diagram with median and interquartile range. **$p \leq 0.01$, two-way ANOVA with Bonferroni correction for multiple comparisons. **(F)** Representative dot plots and **(G)** frequencies (right panel) of splenic plasma cells ($B220^{low}$ $CD138^{+}$) and NP-specific isotype-switched germinal centre B cells (IgM^{-} IgD^{-} $Gr1^{-}$ $CD138^{-}$ $B220^{+}$ NP^{+} $IgG1^{+}$) gated as in Supplementary Figure 9b. ***$p \leq 0.001$, ****$p \leq 0.0001$, two-way ANOVA with Bonferroni correction for multiple comparisons. **(H)** Frequencies of total NP-

specific IgG1-secreting cells in sorted splenocytes as determined by ELISPOT assay. Each dot represents data from a single mouse as mean of results from assays in performed in duplicate from two independent experiments and three mice per experiment (n=6). Data are presented as scatter plot with mean. **$p < 0.01$, GFP+ versus GFP- subsets, two samples Kolmogorov–Smirnov test.

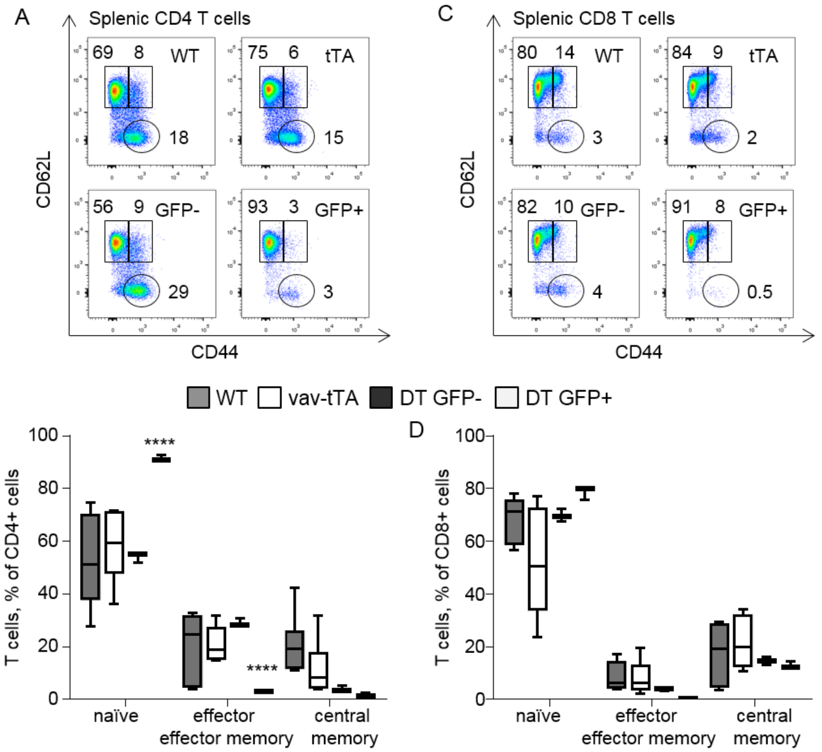

Supplementary Figure 7: tTA expression affects T cell homeostasis after immunization

Flow cytometric analysis of splenic T cells from wild-type, vav-tTA TRE_Ren/vav-tTA double transgenic (DT) mice analysed at day 7 after immunization with NP-KLH. (**A**) Representative dot plots and **(B)** quantification of naïve ($CD4^+$ $CD62L^{high}$ $CD44^-$), effector and effector memory ($CD4^+$ $CD62L^-$ $CD44^-$) and central memory ($CD4^+$ $CD62L^{high}$ $CD44^+$) $CD4^+$ T cells. **(C**) Representative dot plots and **(D)** quantification of naïve ($CD8^+$ $CD62L^{high}$ $CD44^-$), effector and effector memory ($CD8^+$ $CD62L^-$ $CD44^-$) and central memory ($CD8^+$ $CD62L^{high}$ $CD44^+$) $CD8^+$ T cells. Data are presented as box-and-whiskers-diagram with median and interquartile range (n=7). ****p ≤ 0.0001, GFP+ versus GFP-, vav-tTA or wild type controls, two-way ANOVA with Bonferroni correction for multiple comparisons.

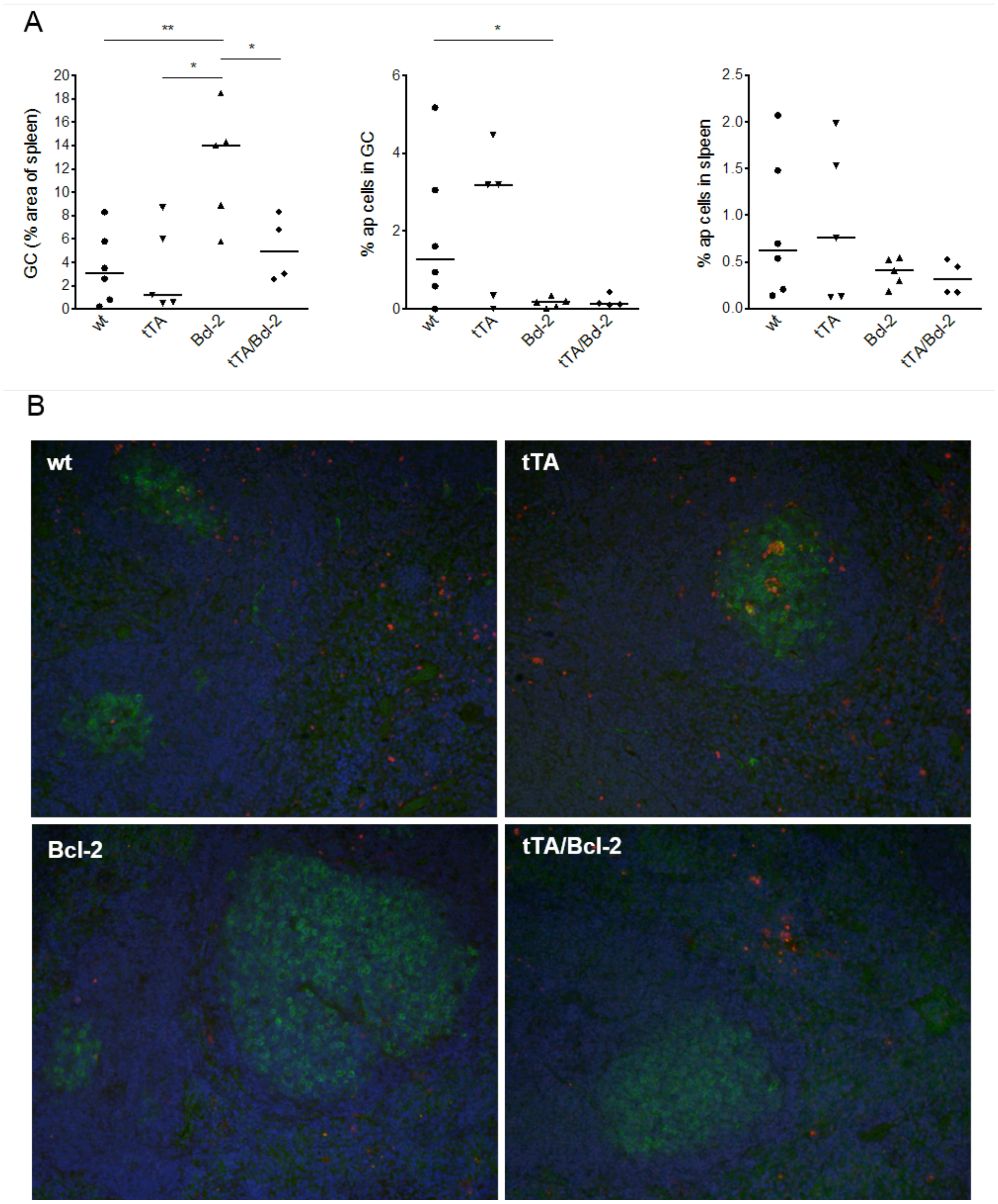

Supplementary Figure 8: Assessment of apoptosis in spleen and GC after immunization

Splenic sections of mice of the indicated genotypes were stained using PNA-FITC to define GC area over total spleen. TUNEL staining was performed using TRITC-labelled dUTP and DAPI counterstaining. Sections were analysed by a blinded observer using the TissueQuest imaging software. Each point represents data from one animal immunized with NP-KLH and sacrificed on day 7. Horizontal bars indicate median values. P-Values have been calculated using the Mann-Whitney U test.

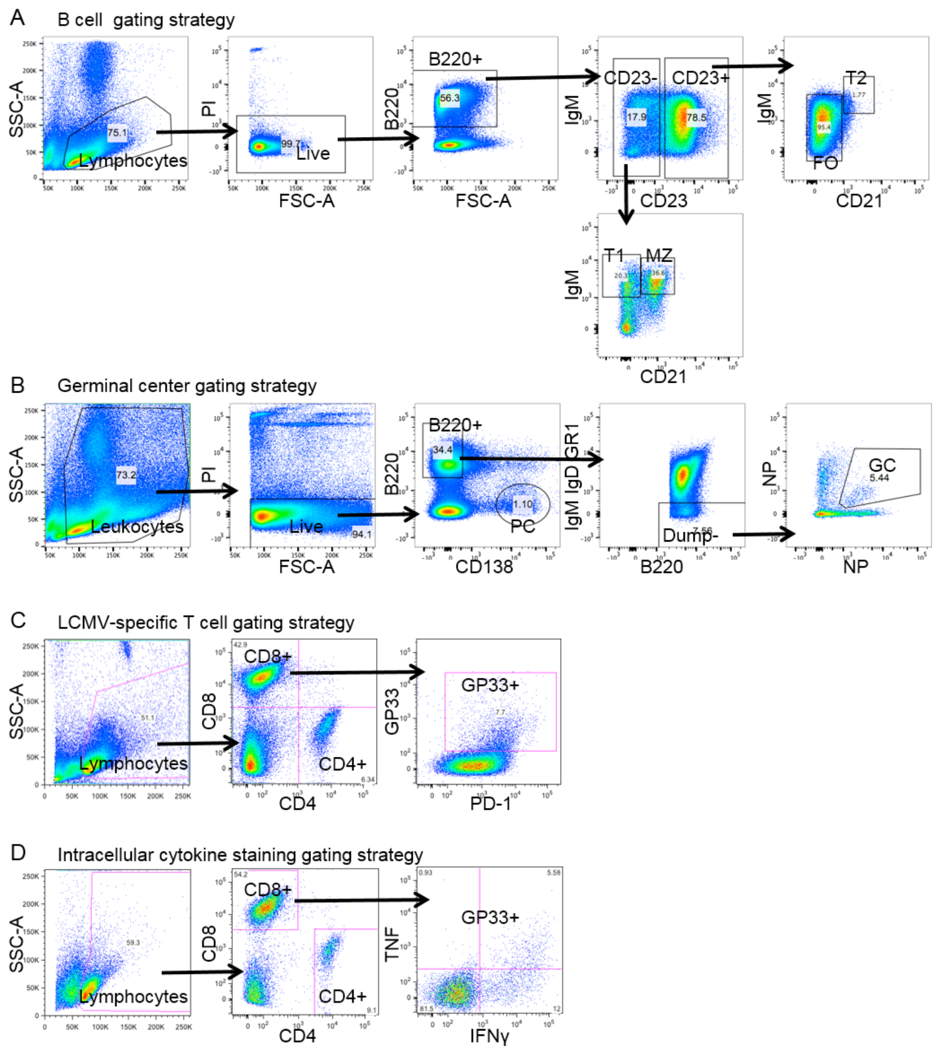

Supplementary Figure 9: Gating strategy used in flow cytometry analysis to detect different immune cell subsets.

(A) Representative dot plots and gating strategy to define transitional type 1 (T1) B cells (B220^{high} IgM^{high} CD23^{low} CD21^{low}), transitional type 2 (T2) B cells (B220^{high} IgM^{high} CD23^{+} CD21^{high}), follicular (FO) B cells (B220^{high} IgM^{low} CD23^{+} CD21^{low}) and marginal zone B cells (B220^{high} IgM^{high} CD23^{low} CD21^{high}). **(B)** Representative dot plots and gating strategy to identify splenic plasma cells (B220^{low} CD138^{+}) and NP-specific isotype-switched germinal centre B cells (IgM^{-} IgD^{-} Gr1^{-} CD138^{-} B220^{+} NP^{+} IgG1^{+}). **(C)** Representative dot plots and gating strategy for LCMV-specific CD8^{+} T cells (CD8+PD-1+ H-2Db/GP33(KAVYNFATM)$^{+}$). **(D)** Representative dot plots and gating strategy for IFNγ and TNF production in virus-specific CD8^{+} T cells after *in vitro* restimulation of splenocytes with the cognate peptide for 4 hours.

Supplementary Table 1. Antibodies used in this study.

Antibody	Vendor	Clone	Catalouge no.	Dilution
CD38	Biolegend	NIMR-5	102712	1/200
B220	eBioscience	RA3-6B2	48-0452-80	1/400
IgM	Biolegend	RMM-1	406523	1/800
IgD	Biolegend	11-26c.2a	405727	1/800
Gr-1	Biolegend	RB6-8C5	108439	1/400
CD138	BD Pharmingen	281.2	553714	1/1000
IgG1	BD Pharmingen	X56	550874	1/600
FcγR	eBioscience	2.4G2	12-0161-82	1/200
PNA	Sigma	FL-1071	L7381	1/200
CD21	Biolegend	7 E9	123411	1/200
CD23	Biolegend	B3B4	101611	1/800
CD4	Biolegend	RM4-5	100527	1/1000
CD8	Biolegend	53-6.7	100733	1/400
PD-1	Biolegend	J43	135219	1/800
CD62L	Biolegend	MEL-14	104411	1/500
CD44	BD Pharmingen	IM7	563114	1/100
CD69	eBioscience	H1.2F3	48-0691-80	1/100
CD11c	Biolegend	HL3	117312	1/100
CD11b	Biolegend	M1/70	101225	1/200
CD317	eBioscience	297	127015	1/200
NP	Biosearch Technologies		N-5070-1	1/400
IFNϒ	Biolegend	XMG1.2	505816	1/50
TNF	eBioscience	MP6-XT22	17-7321-41	1/200
IgG1-HRPO	Southern Biotech	antisera	1020-05	1/1000
